# Investigation of post-translational modifications in type 2 diabetes

**DOI:** 10.1186/s12014-018-9208-y

**Published:** 2018-09-24

**Authors:** Bhaswati Chatterjee, Suman S. Thakur

**Affiliations:** 1 Department of Pharmaceuticals, Ministry of Chemicals and Fertilizers, Govt. of India, National Institute of Pharmaceutical Education and Research (NIPER), Balanagar, Hyderabad, Telangana 500 037 India; 2 Proteomics and Cell Signaling, Lab E409, Centre for Cellular and Molecular Biology, Uppal Road, Hyderabad, 500007 India

**Keywords:** Post-translational modifications, Type 2 diabetes, N-glycosylation, O-GlcNAcylation, Acetylation, Phosphorylation, High throughput mass spectrometry based proteomics

## Abstract

The investigation of post-translational modifications (PTMs) plays an important role for the study of type 2 diabetes. The importance of PTMs has been realized with the advancement of analytical techniques. The challenging detection and analysis of post-translational modifications is eased by different enrichment methods and by high throughput mass spectrometry based proteomics studies. This technology along with different quantitation methods provide accurate knowledge about the changes happening in disease conditions as well as in normal conditions. In this review, we have discussed PTMs such as phosphorylation, N-glycosylation, O-GlcNAcylation, acetylation and advanced glycation end products in type 2 diabetes which have been characterized by high throughput mass spectrometry based proteomics analysis.

## Background

Type 2 diabetes has become a global health problem as the diabetic population is estimated to increase to 552 million by the year of 2030 [1]. This metabolic disorder involves a progressive loss in the body’s ability to respond to insulin (insulin resistance) resulting in hyperglycemia and the pancreas compensates for this loss causing hyperinsulinemia. Eventually, the overworking pancreas loses the capacity to produce insulin. In type 2 diabetes, the death of beta cells is triggered by nutrients via NF-kappa B-independent mechanism [2]. Insulin resistance is the best predictor of type 2 diabetes. The insulin resistance and type 2 diabetes mellitus is associated with decreased expression of multiple nuclear respiratory factor-1 (NRF-1)-dependent genes that may be due to decreased expression of peroxisomal proliferator activator receptor γ coactivator (PGC1) [3]. Interestingly, the sleep disorder is also associated with insulin resistance and type 2 diabetes [4]. Insulin resistance occurs in obese people, type II diabetes, lipodystrophy condition due to the change in division of fat between adipocyte and liver/muscle. This leads to the deposition of triglycerides, fatty acid metabolites in insulin responding cells/tissues leading to defects in insulin signalling and hence insulin resistance [5]. Insulin resistance in skeletal muscle may be caused due to defect in glucose transporter GLUT-4 [6]. In the type 2 diabetes patients, adipose tissue showed insulin resistance by having defect in glucose transport [7] and low insulin receptor kinase activity [8]. Another interesting contributor towards insulin resistance and type 2 diabetes is the ectopic deposition of lipids inside non-adipose tissues [8–10].

Hyperinsulinemia is able to increase IRS-1 tyrosine phosphorylation and PI3-kinase function in normal human skeletal muscle compared to skeletal muscle of non-insulin-dependent diabetic subjects [11].

The activity of most of the proteins in type 2 diabetes is modulated by the presence of post-translational modifications (PTMs). There is great deal of challenge associated with detection and characterization of these post-translational modification and studies on the PTMs throws great deal of light into the biological activities of the proteins. The PTMs are required for cell signaling process.

The non-enzymatic PTMs that are uncontrolled have harmful effect on proteins such as glycation which causes changes in conformation of proteins, form aggregates and Maillard cross-links [12]. The oxidative and the enzymatic post-translational modification are involved in the autoimmune diseases such as rheumatoid arthritis, systemic lupus erythematosus, type-1 diabetes mellitus and others [13]. In the patients with type-I diabetes having HLA-DRB1*4 allele associated rheumatoid arthritis, the Type-II collagen antigenicity is increased significantly by oxidative PTMs while its increase is less in type-I diabetes patients without HLA-DRB1*4 allele associated rheumatoid arthritis [14].

The misfolding of proteins due to the contribution from PTMs is attracting attention also in the field of proteopathies [15, 16]. The Type II diabetes mellitus is a protein conformation disorder because of the formation of beta-pleated sheets thereby aggregation resulting change in the tertiary structure of islet amyloid polypeptide proceeded by self-association and deposition of tissues [17].

This review discusses different post-translational modifications that have been found in type 2 diabetes characterized by high throughput mass spectrometry based proteomics.

## Proteomics technologies

The proteomic technologies are for identification and quantification of proteins and different post-translational modifications in proteins present in cells, tissues or an organism. The proteins are digested into peptides that are amenable to high resolution mass spectrometry based analysis. Before their identification and characterization by mass spectrometry, they are subjected to different enrichment methods for specific post-translational modifications that are described under different post-translational modifications.

### Mass spectrometry

The choosing of the technology depends upon the aim of study and its availability. The most commonly used mass spectrometry for proteomics among other mass spectrometry [18] are those having quadrupole time-of-flight (TOF) and hybrid linear ion trap–orbitrap configurations. The configurations of these instruments combine sensitivity, speed, and robustness with high resolution capabilities. The TOF instruments have resolution around 10,000 [19], the LTQ Orbitrap has resolution of 60,000 [20] while Orbitrap Elite has resolution of 240,000 [21]. The standard orbitrap mass analyzer has inner diameter of outer electrode 30 mm while the same for high field orbitrap mass analyzer (Orbitrap Elite) is 20 mm. In Orbitrap Elite, the size of the inner electrode is decreased to 10 mm from 12 mm which is of standard trap mass analyzer [21]. The mass accuracy for the identified peptides is in parts per million in TOF while it decreases further to become parts per billion in orbitrap [22]. The quantification of complexed proteins/peptides in very less amount is also done by using PCT-SWATH [23]. The SWATH-MS combines data-independent acquisition with data analysis, analogous to S/MRM. This is helpful in case of clinical samples where immediately after sample collection, SWATH maps are generated for each of the patient sample giving a quantitative proteomic digital profile of the individual [23].

### Type of fragmentations

The most common way to fragment peptide ion is collision-induced dissociation (CID) that involves the collision of peptide or precursor ion with inert gas at low pressure [24, 25]. The peptide ions are accelerated by electric field to high kinetic energy and some of this kinetic energy is converted to internal energy after their collision leading to fragmentation at the peptide bond in the ion trap. The fragmentation in ion trap is of low resolution leading to the formation of many uninformative ions. The information about uninformative ions can be decoded using sophisticated ion traps [26]. In quadrupole TOF machine, mass filters are present that helps in the transmission of precursor ion of interest, leading to their fragmentation by CID in the collision cell. In this more informative ions are detected due to presence of more sensitive TOF analyzer. Another fragmentation technique is higher energy collisional dissociation (HCD) where the radiofrequency voltage is increased to 2500 V for retaining the maximum number of fragment ions in the C-trap with the increase in y ions [20, 27]. The other fragmentation mode involves electron capture dissociation (ECD) and electron transfer dissociation (ETD) that involves different fragment ions by involvement of electrons thereby retaining labile post-translational modifications [28–31].

### Type of quantitation/labeling techniques

One of the potential of mass spectrometry over traditional methods is the ability to quantitate accurately large number of proteins and its post-translational modifications. For highly accurate quantitative results, the two proteomes have to be compared one labelled and other non-labeled. One of the metabolic labelling methods is labeling with Stable isotope labelling by amino acids in cell culture (SIlAC) [32–34], where heavy non-radioactive isotope of Lysine and Arginine amino acids containing ^13^C isotope and/or ^15^N isotope is used. Both light (normal) and heavy (labelled) Lysine and Arginine is used in cell culture for the growth of two sets of cell populations. Here the cells incorporate these heavy isotopes as part of normal biosynthesis. The carboxylic end of the peptide contains a labeled amino acid after digestion with trypsin. The know molecular weight difference between the light and heavy isotope of Lysine and Arginine helps in identifying and quantifying the proteins. Triple SILAC labelling uses addition label of ^13^C_6_ Arginine and D-labelled Lysine thereby helping in comparison between three states proteomes and measurements involving time points. This technology is extended also to human tissue sample [35] and also in whole organism [36–38]. This isotope labeling technology can be used to quantifying proteomes in cultured insulin secreting cell lines RIN, HIT, beta-TC, MIN6 and INS-1 cells that help to study insulin secretion and cell dysfunction [39]. Apart from SILAC, metabolic labeling by ^15^N has been used in *C. elegans* and *D. melanogaster* by feeding the respective organism by ^15^N labeled *E*. *coli* and yeast [40]. Super-SILAC is the extension of SILAC technology involving combination of five SILAC cell lines with the tissue sample generating lots of labelled peptides that may be used as internal standard for analysis involving mass spectrometry [35].

There are many chemical labeling techniques that are used to quantify the proteome. One of the disadvantages of chemical labeling is side products that formed during chemical derivatization leading to the suppression in the identification rate of the proteins and presence of artifacts [41]. The chemical labeling techniques at peptide level like stable isotope dimethyl labeling [42, 43] is cost-effective and applicable to any diabetic sample with amounts varying from sub-micrograms to milligrams. The isobaric tag for relative and absolute quantification (iTRAQ) is another popular chemical labeling technique where there is covalent attached of labelled group to the amine group at the N-terminus and at the side chain of the amino acids at the peptide level [44]. This labeling technique is observed after the fragmentation of the precursor ion with the elimination of co-fragmented ions having similar masses and same retention time [45, 46]. An alternative form of protein labeling followed by LC–MS/MS is Isotope-coded affinity tags (ICAT) that uses biotin, stable isotope signatures linker and reactive reagent having affinity for free thiol group [47].

Sometimes label-free quantitation offers the benefit where there is no experimental manipulation at the protein and at peptide levels [48] and offers the successful comparison of the normalized peptide signals under different experimental conditions [49, 50]. Even though there is limitation in its analysis, it has been successfully used by advancement in its computational approaches [51].

There have also been cases where absolute quantitation of proteins is required to determine their expression levels. The absolute quantification (AQUA) is one such method involving the use of known concentration of synthetic internal standard peptide with stable isotopes that mimics a peptide produced during the digestion of the target protein followed by analysis by selected reaction monitoring (SRM) in LC–MS/MS [52]. Another method, Absolute SILAC was also used for accurate quantitation of selected proteins in a complex mixture [53].

### Computational analysis of proteomics data

Analysis of proteomic data with the help of mass spectrometry involves extraction of signals from the peptides, identification of peptides with help of search engines to enable the identification of proteins and then protein quantification with false positive rate usually set to 1% [54]. The quantification of proteins becomes important if it is set at a specific p value [22]. The peptide-centric scoring tool obtained from SWATH-MS data provides consistent and accurate quantitative proteomics data [55]. After quantification, there involves bioinformatics approach involving gene ontology enrichment that tells about cellular compartments, cellular functions or KEGG pathway mapping [56, 57]. The great challenge in the field of proteomics is the integration and data mining of these huge dataset.

## Post-translational modifications involved in type 2 diabetes

The importance of PTMs in the biological activity has been realized with the advancement of analytical techniques. Most of the therapeutic proteins approved or those in clinical trials have PTMs that affect the properties of the proteins, their stability that are important for therapeutic purposes. The post-translation modified peptides can be detected with mass spectrometry as it is very sensitive in the change of mass of the modified peptides along with the localization of the modification in particular amino acid. One of the challenges in mass spectrometry based proteomics is the detection and identifications of PTMs that are present at low stoichiometry’s thus necessitating the role of enrichments methods. There are many enrichment methods each specific for different PTMs as there are variety of protein modifications. Table 1 list the most studied PTMs in diabetes amenable to large scale mass spectrometry based analysis. It tells about the highest number of detectible PTM sites by high throughput mass spectrometry based proteomics analysis with the help of different enrichment methods.

**Table 1:** Post-translational modifications compatible to high throughput mass spectrometry based proteomics analysis in diabetes

Post-translational modifications	ΔMass^a^ (monoisotopic mass) in Da	Largest MS dataset^b^ sites/peptides	Organisms	Enrichment mode
Phosphorylation	79.96633 [129]	12,294 sites [76]	Rat hepatocytes	TiO_2_ beads
Acetylation	42.01056 [129]	1604 sites [123]	Mouse liver	Polyclonal acetyl-lysine K(ac) antibody
O-GlcNAcylation	203.07937 [129]	1750 sites [99]	Synaptosome (mouse)	Lectin wheat germ agglutinin column
N-Glycosylation	HexNAcoxonium ions (138.0545 and 204.0867), deamidation of asparagine and glutamine (0.9848)	951 unique deamidation sites, 1580 unique N-glycopeptides [90]	3T3-L1 adipocytes/fibroblasts (mouse)	zic-HILIC SPE column, PNGase F
Advanced glycation end products	Amadori compound modification at lysine (162.0528 Da)	7749 unique glycated peptides [124]	Diabetes human plasma and erythrocytes	Boronate affinity chromatography

Largest post-translational modified sites detected using mass spectrometry from different sources by different enrichment methods

*MS* mass spectrometry

^a^Reference for ΔMass

^b^Reference to the largest proteomics dataset for each post-translational modifications

### Phosphorylation

In mass spectrometry, the identification of phosphorylation in proteins becomes tricky due to low ionization, fragmentation and stoichiometry of phosphopeptides. To overcome these problem many techniques were applied for analysis of phosphopeptides. The enrichment techniques for phospho-peptides which helps them to separate from non-phosphopeptides involves the immobilized metal affinity chromatography (IMAC) based on the coordination of phosphates to certain metal ions such as Ga^3+^, Fe^3+^, Zr^4+^ and Ti^4+^ [58–62], complex of titanium dioxide-2,5 dihydroxybenzoic acid [63], hydrophilic interaction chromatography (HILIC) [64], strong cation exchange (SCX) [65], immunopurification (IP) with immobilized anti-phosphotyrosine antibodies [66–69].

The high throughput mass spectrometry studies have identified majority of protein phosphorylation occurring on serine and threonine residues compared to tyrosine residue. Interestingly, serine: threonine: tyrosine phosphorylation ratios were found to be 90:10:0.05 based on autoradiography measurements [70]. The problem associated in using phosphoproteomics in human diabetes research is less availability of human pancreatic islets samples compared to mouse samples or cell line studies.

Protein phosphorylation is important for the proper functioning of pancreatic β-cell. The pancreatic β-cells are the production house for insulin and are one of the factors for the healthy survival. When the β-cells of pancreas are stressed, they undergo dedifferentiation and dysfunction where they produce very less amount or no insulin [71, 72]. Loss of two Ins2 and Ins1 alleles in adult mouse β-cells causes loss of insulin production resulting in hyperglycemia. This is accompanied with increase of β-cells proliferations and decrease of stress in endoplasmic reticulum. This leads to low expression of Ddit3, Trib3, Xbp1 splicing, phospho-elF2α and Atf4 with an over phosphorylation of Akt at serine 473 along with upregulation of cyclinD1. Moreover CyclinD1 is known to be a key player in post-natal proliferation of β-cells. The increase in phosphorylation of Akt is due to combine effect of Hdac2, Crk and Set/Nme1 apart from Trib3. The hyperactivity of Akt is enough to increase proliferations of β-cells via cyclinD1 [73]. Phosphorylation plays an important role in glucose stimulated insulin secretion in islets of Langerhans and serves as important mediator in insulin stimulated signaling networks. An in-depth mapping of phosphoproteome with the help of phosphopeptide enrichment studies done with titanium dioxide and SILAC strategy revealed the presence of 8539 phosphosites on 2487 proteins (Table 1) with glucose stimulated rat pancreas islets. Furthermore, detection of phospho-Serine 89%, phospho-Threonine 10% and phospho-Tyrosine 1% sites were reported. This lead to identification and confirmation of glucose-responsive phosphosites such as Prkar1a phospho-Threonine 75, phospho-Serine 77 and Tagln2 phospho-Serine 163 related with insulin secretion. The level of phosphorylation was found to be up-regulated in case of Prkar1a Threonine 75 and Serine 77 while down-regulation was seen in case of Tagln2 Serine 163 [74].

The different cell types have varying insulin signaling pathways [75]. On stimulation with insulin in primary rat hepatocytes resulted in activation of PI3K-Akt-mTORC1-S6K pathway. Interestingly, the reductive demethylation using heavy and light isotopes of formaldehyde at the peptide level was used to study 3805 phosphoproteome with 12,294 unique phosphosites regulated by insulin. The serine residue constitute 81.6% phosphorylation, while threonine and tyrosine residues have 15.3% and 3.2% phosphorylation respectively. Further, the identification of phospho-tyrosine sites (tyrosine 650, tyrosine 672 and tyrosine 735) on IRS2 upon stimulation with insulin leads to association of IRS2 with PI3K, activation of Akt and uptake of glucose [76]. An increase of tyrosine phosphorylation for iTRAQ quantified phosphopeptides including changes in CD4^+^ T cell compartments signaling have been found in non-obese diabetes (NOD) mice compared to that of diabetic-resistant B6g7 mice suggesting dysregulation of T cell receptor signaling in NOD mice. These phosphopeptides were detected using immunoprecipitation by anti-phosphotyrosine antibody, and IMAC enrichment [77].

The disturbance in phosphorylation of ATP synthase beta subunit (ATPsyn-β) results in dysregulation of ATP synthesis along with decreased level of OxPhos proteins. This may lead to development of insulin resistance and type 2 diabetes. In obesity and type 2 diabetes there is increased phosphorylation at threonine 213 and tyrosine 361 sites of ATPsyn-β. Incidentally the activity of ATP synthase is decreased due to phosphorylation at serine residue of ATPsyn-β [78]. Increase of insulin stimulates the synthesis of fatty acids in liver. Insulin resistance and obesity induced by diet in mice is ameliorated by blockage of Casein Kinase (CK2) pathway resulting in biogenesis of beige adipocyte. The CK2, a serine/threonine kinase pathway is more activated in white adipocytes than in beige/brown adipocytes. In white adipocytes, nor-ephinephrine stimulates CK2 while inhibition of CK2 results in cAMP-induced thermo genesis with reduction in phosphorylated class I HDACs especially phosphorylated serine 393, 421, and 423 sites of HDAC1 along with phosphorylated serine 422 and 424 sites of HDAC2 [79].

In skeletal muscle, the serine/threonine protein kinase 25 (STK25) is known to aggravate insulin resistance. Moreover, in skeletal muscle, the overexpression of STK25 increases the storage of fat and deregulates the mitochondrial function in mice. Notably, phosphoproteomics studies suggest alterations of phosphorylated proteins mainly in mitochondria and sarcomeric contractile muscle thereby identifying mediators of the action of STK25 [80].

### Glycosylation

The O-GlcNAc and N-glycosylation modifications is a sensor linking cellular metabolism with various signaling pathways and is associated in the pathogenesis of diabetes [81] as these modification increases with hyperglycemia. Investigation of glycosylation is difficult owing to variation in the structure of glycans. This modification due to the presence of glycosidic bond is very labile and largely goes undetected in mass spectrometry due to ion suppression in the presence of unmodified peptides. With the development of different ionization techniques, electron capture dissociation (ECD) and electron transfer dissociation (ETD) have improved the site-mapping ability of O-GlcNAcylation and N-glycosylation modification [82, 83]. There is need for the enrichment of O-GlcNAcylated/N-glycosylated peptides or proteins before mass spectrometry studies so as to avoid the suppression of O-GlcNAcylated/N-glycosylated peptides or proteins in the presence of unmodified peptides or proteins. Notably, several useful methods are available for the separation of glycopeptides from non-glycopeptides. For enrichment of O-glycopeptides, the hydrophilic interaction chromatography (HILIC) column is not efficient as O-glycans are smaller in size than N-linked glycans. It has been found that before the enrichment of O-linked glycopeptides, it is fruitful to remove N-linked glycopeptides by PNGase F. The strong anion exchange (SAX) chromatography has been found to be useful for enrichment and identification of N- and O-linked glycopeptides. Further improvement in enrichment of O-linked glycopeptides was obtained with the use of Retain AX cartridge (RAX) [84]. The other interesting chromatographic methods involving the separation of non-glycopeptides from glycopeptides involves the combined usage of lectin-affinity chromatography and polysaccharide hydrophilic affinity physicochemical chromatography [85].

### N-glycosylation

N-glycosylation is the covalent bonding of glycans to the N-linked Asn residue. The enrichment techniques for N-glycosylated proteins or peptides involves various methods such as affinity with lectin [86], hydrazide chemistry linking sugar residue to surfaces [87], magnetic colloidal nanocrystal clusters coated with chitosan [88] and Zwitterionic hydrophilic interaction chromatography with solid-phase extraction (ZIC-HILIC SPE) [89].

Insulin resistance is also linked with TNF-alpha. A quantitative study in SILAC labelled mouse 3T3-L1 adipocytes cells incubated with TNF-alpha revealed the presence of 1580 unique N-glycopeptides (Table 1) with an increase in di-galactose, glycosyltransferases-B4GalT5 and Ggta1 and decrease in alpha-2, 3 sialoglycans, ST3Gal6 sialyl transferase by enrichment with zic-hydrophilic interaction liquid chromatography solid phase extraction column column (HILIC SPE). This study also shows that galactosyltransferase-B4GalT5 is involved in regulation of N-glycome by TNF-alpha. Moreover, it revealed the changes in N-glycosylation on important proteins involved in insulin resistance including GLUT4 [90]. A similar study on insulin resistance was carried out by inducing insulin resistance in primary human adipocytes revealing 91 N-glycosylation sites with 155 and 29 N- and O-glycans respectively. This supports that impairment of adipo-cytokines leads to insulin resistance and type 2 diabetes mellitus with increase in global O-linked β-N-acetylglucosamine (O-GlcNAc) levels [91].

### O-GlcNAcylation

O-GlcNAcylation is the covalent bonding of single O-linked N-acetyl-β-D-glucosamine (O-GlcNAc) group to the hydroxyl group of serine and threonine residues. The O-GlcNAcylated peptides or proteins can be enriched before mass spectrometry studies. This enrichment involves various methods including CTD110.6 anti-O-GlcNAc mouse monoclonal antibody [92], O-GlcNAc-specific IgG monoclonal antibodies [93], enzymes that cause the addition and removal of O-GlcNAc [94], site-specific antibodies [95–97], affinity chromatography with GlcNAc-binding lectin, wheat germ agglutinin [98, 99], cells labelled by azide-modified GlcNAc with click chemistry mediated by copper [100] and in vitro sulfation [101].

The O-GlcNAcylation is stimulated by high level of glucose through hexosamine biosynthetic pathway (HBP) [102]. With the increase of O-GlcNAcylation levels there is damage to pancreatic β cell and this is proved by exposing these cells to streptozotocin and/or glucosamine thereby enhancing the relationship between HBP and diabetes [103]. The changes in HBP are a major factor for insulin resistance, and is not only related to glucose but also to glutamine and the pentose phosphate pathway. Increase of O-GlcNAcylation through HBP results in abnormal O-GlcNAcylation of proteins involved in cytoskeleton including α-actinin 4, actin (serine 201 residue) and myosin (serine 1038 residue) thereby causing changes in morphology of glomerulus and tubules in diabetic kidney. Notably, in diabetic kidney, the level of O-GlcNAcylation is increased in α-actin, α-tubulin, α-actinin 4, myosin proteins that are part of cytoskeletal proteins along with ATP synthase β and pyruvate carboxylase that being mitochondrial proteins. [104].

Elevated O-GlcNAc not only effects kidney cells, it also effects cardiac [105], liver and skeletal muscles. The increase of hyperglycemia leads to covalent attachment of O-GlcNAc to Ser 279 of Ca^2+^/calmodulin-dependent protein kinase II (CaMKII) thereby activating it and creating a feeling of the presence of Ca^2+^ even though its concentration is less leading to cardiac dysfunction and arrhythmias [106]. The O-GlcNAc enrichment was done using beta-elimination proceeded by Michael addition with dithiothreitol. Interestingly, removal of excess of specific O-GlcNAc from diabetic myo filaments improves cardiac functions and restores its Ca^2+^ sensitivity [107]. Moreover, elevated O-GlcNAcylation in hyperglycemia causes mitochondrial dys-functioning in heart [108].

Upon insulin resistance, Insulin receptor substrate-1 (IRS-1) an important protein in insulin signaling pathway, was found to be O-GlcNAc modified by using β-elimination followed by Michael addition with DTT (BEMAD) prior to LC–MS/MS thereby suggesting important role of this modification site in insulin signaling by inhibiting phosphorylation at these sites [109]. This modification on IRS-1 occurs very close to multiple SH2 domain binding motifs suggesting interaction among them [110].

The O-(2-acetamido-2-deoxy-d-glucopyranosylidene) amino-N-phenylcarbamate (PUGNAc) is an inhibitor of O-GlcNAcase leading to insulin resistance [111] as seen in the studies where insulin resistance is caused with higher levels of O-GlcNAcylation in 3T3-L1 adipocytes [112] and in rat skeletal muscle [113] treated with PUGNAc. Using PUGNAc, ATP affinity chromatography and targeted proteomic approach in 3T3-L1 adipocytes it was that found there was increase of O-GlcNAcylation and ubiquitination of proteins with the decrease in level of Akt and its phosphorylation, with Hsp90 down-regulation showing the possible inhibition of insulin signaling [114].

Interestingly, transcription factors and co-activators including FoxO1, PGC-1α, CRTC2 that play an important role in gluconeogenesis are O-GlcNAcylated under high glucose conditions resulting in activation of proteins such as phospho enol pyruvate carboxykinase and glucose 6-phosphate involved in gluconeogenesis [115–117]. In diabetes mice, hepatic knockdown of O-GlcNAc transferase (OGT) and host cell factor C1 (HCF-1) results in improvement of homeostasis of glucose confirming these as sensor for glucose and gluconeogenesis regulator [117].

The O-GlcNAc modified human erythrocyte proteins were found to be up-regulated and potential biomarker for diabetes and pre-diabetes, based on chemo enzymatic tagging with quantitative mass spectrometry. This led to identification and quantification of O-GlcNAc sites suggesting the development of targeted site specific antibodies will help in identifying pre-diabetes patients [118].

### Acetylation

Acetylation in proteins occurs typically in lysine residues as it has two active hydrogens in NH group at its side chain. Due to presence of two active hydrogens in NH group it can be mono-acetylated and di-acetylated. Most commonly, mono-acetylation occurs in the lysine residues of proteins. It is a reversible post-translational modification and is dependent on acetylases and deacetylases enzymes for its acetylation and de-acetylation.

Acetylome plays an important role in understanding the physiological difference between Goto-Kakizaki (GK) rats and normoglycemic Brown Norway (BN) rats. The difference was the down-regulation of Sirtuin-3 by promotor SNP in GK rat [119].

Increase of acetylation on lysine residue in retinal histones histone H3 and H4 in diabetes leads to regulation of pro-inflammatory proteins activities in diabetic retinas suggesting their contribution towards the pathogenesis of the disease [120]. Similarly, increase in lysine acetylation in heart mitochondrial proteins in type 1 diabetes damages the insulin signaling pathway with the increase in fatty acid oxidation [121]. The dual effect of defect in mitochondrial complex 1 and amplification in fatty acid oxidation incremented the lysine acetylation in diabetes mitochondria with no change in level of lysine deacetylase, sirt3. The defect in mitochondrial complex 1 leads to decrease in activity of reduction of coenzyme Q, NADH dehydrogenase, oxidation of ferricyanide and oxidation of NADH [122].

Another quantitative global analysis of the liver of diabetes mice led to the mapping of 1604 acetylation sites (Table 1) by enrichment with polyclonal acetyl-lysine K(ac) antibody. Interestingly, more number of acetyl-lysine was found in mitochondrial proteins involved in urea cycle and in conversion of amino acids and energy metabolism. The presence of acetylated peptides in several pathways suggested its comparability with phosphorylation of serine and threonine residues [123].

### Advanced glycation end products (AGEs)

An AGEs is the non-enzymatic covalent bonding of sugars such as glucose or fructose to the free amino or guanidinium group of proteins, lipids and nucleic acids. The initial product is Schiff base which rearranges itself to Amadori product. The Amadori product degrades giving rise to AGEs. The compounds that are widely studied as examples of AGEs are N-carboxymethyl-lysine, N-carboxyethyl-lysine, pentosidine, or methylglyoxal derivatives, pyrraline, imidazolone, lysine–lysine cross-links. By boronate affinity chromatography enrichment studies, 7749 highest unique glycated peptides were identified in diabetes plasma and erythrocytes (Table 1) by mass spectrometry based proteomics and it was found erythrocyte proteins were more glycated than plasma proteins in diabetes [124].

The AGEs is an oxidative PTM resulting from oxidative stress disrupting biological activities thereby associating it with several diseases example, diabetes. There are several cell surface receptors for AGEs [125] that mediates different signaling pathways activated by AGEs for example multi-ligand receptor for advanced glycation end products (RAGE), AGE-receptor complex (AGE-R1/OST-48, AGE-R2/80K-H, AGE-R3/galectin-3) and scavenger receptor family SR-A, SR-B: CD36, SR-BI SR-E: LOX-1; FEEL-1; FEEL-2. Lactadherin acts as mediator in endothelial cell apoptosis induced by AGEs. The over expression of lactadherin increases the apoptosis of endothelial cells with the upregulation of ratio of Bax/Bcl-2, activation of the release of cytochrome c, caspase-9 and caspase-3 and reduction of phosphorylation of GSK3beta [126].

## Conclusions

Mass spectrometry based proteomics technology has provided an opportunity to study proteins, their PTMs like phosphorylation, acetylation, N-glycosylation, O-GlcNAcylation and Advanced glycation end products in type 2 diabetes. In future, the proteomic technologies will have to be sensitive to detect the concentration of the proteins that are playing an important role in the disease condition or detect the fast turnover of newly synthesized proteins. With the advancement in high resolution mass spectrometry and with the combination of good quality sample preparation, enrichment techniques along with improvement of computational tools one can study large number of PTMs that modify the signaling pathways in type 2 diabetes. Development of mass spectrometry especially quantitative and targeted proteomics will be of great alternate to antibody validation. Furthermore, the advancement in proteomics technology will help in detection and studying the cross-talk between multiple low abundant PTMs thereby enabling to get further insight into type 2 diabetes.

Most of the proteins involved in type 2 diabetes have PTMs that affect the properties of the proteins, their stability and may serve as targets for therapeutic purposes. A good understanding of structure–function relationship of the PTMs is required for the generation of products of therapeutic importance [127] and also for understanding the progression and development of type 2 diabetes. The PTMs present a large number of candidates for biomarker discovery in type 2 diabetes. However, there are many challenges associated with the flourishing implementation of proteomic biomarker discovery in medical practice. This can be resolved by involving wide range of stakeholders so as to bring biomarker discovery to healthcare [128].

## References

[1] International Diabetes Federation. International Diabetes Federation’s Diabetes Atlas, 5th ed, 2011.

[2] Cnop M, Welsh N, Jonas JC, Jörns A, Lenzen S, Eizirik DL (2005). Mechanisms of pancreatic beta-cell death in type 1 and type 2 diabetes: many differences, few similarities. Diabetes.

[3] Patti ME, Butte AJ, Crunkhorn S, Cusi K, Berria R, Kashyap S, Miyazaki Y, Kohane I, Costello M, Saccone R, Landaker EJ, Goldfine AB, Mun E, DeFronzo R, Finlayson J, Kahn CR, Mandarino LJ (2003). Coordinated reduction of genes of oxidative metabolism in humans with insulin resistance and diabetes: potential role of PGC1 and NRF1. Proc Natl Acad Sci USA.

[4] Spiegel K, Knutson K, Leproult R, Tasali E, Van Cauter E (2005). Sleep loss: a novel risk factor for insulin resistance and type 2 diabetes. J Appl Physiol.

[5] Shulman G (2000). Cellular mechanisms of insulin resistance. J Clin Invest..

[6] Shepherd PR, Kahn BB (1999). Glucose transporters and insulin action—implications for insulin resistance and diabetes mellitus. N Engl J Med.

[7] Lonnroth P, Digirolamo M, Krotkiewski M, Smith U (1983). Insulin binding and responsiveness in fat cells from patients with reduced glucose tolerance and type II diabetes. Diabetes.

[8] Anstee QM, Targher G, Day CP (2013). Progression of NAFLD to diabetes mellitus, cardiovascular disease or cirrhosis. Nat Rev Gastroenterol Hepatol..

[9] Perseghin G, Scifo P, De Cobelli F, Pagliato E, Battezzati A, Arcelloni C, Vanzulli A, Testolin G, Pozza G, Del Maschio A, Luzi L (1999). Intramyocellular triglyceride content is a determinant of in vivo insulin resistance in humans: a 1H-13C nuclear magnetic resonance spectroscopy assessment in offspring of type 2 diabetic parents. Diabetes.

[10] Goodpaster BH, Wolf D (2004). Skeletal muscle lipid accumulation in obesity, insulin resistance, and type 2 diabetes. Pediatr Diabetes..

[11] Björnholm M, Kawano Y, Lehtihet M, Zierath JR (1997). Insulin receptor substrate-1 phosphorylation and phosphatidylinositol 3-kinase activity in skeletal muscle from NIDDM subjects after in vivo insulin stimulation. Diabetes.

[12] Harding JJ, Ganea E (2006). Protection against glycation and similar post-translational modifications of proteins. Biochim Biophys Acta.

[13] Ryan BJ, Nissim A, Winyard PG (2014). Oxidative post-translational modifications and their involvement in the pathogenesis of autoimmune diseases. Redox Biol..

[14] Strollo R, Rizzo P, Spoletini M, Landy R, Hughes C, Ponchel F, Napoli N, Palermo A, Buzzetti R, Pozzilli P, Nissim A (2013). HLA-dependent autoantibodies against post-translationally modified collagen type II in type 1 diabetes mellitus. Diabetologia.

[15] Nilsson MR, Sipe JD (2005). Post-translational chemical modifications in amyloid fibril formation. Amyloid proteins the beta sheet conformation and disease.

[16] Abedini A, Gupta R, Marek P, Meng F, Raleigh DP, Taskent H, Tracz S, Ramirez-Alvarado M, Kelly JW, Dobson CM (2010). Role of posttranslational modifications in amyloid formations. Protein misfolding diseases: current and emerging principles and therapies.

[17] Hayden MR, Tyagi SC, Kerklo MM, Nicolls MR (2005). Type 2 diabetes mellitus as a conformational disease. JOP..

[18] Domon B, Aebersold R (2006). Mass spectrometry and protein analysis. Science.

[19] Chernushevich IV, Loboda AV, Thomson BA (2001). An introduction to quadrupole-time-of-flight mass spectrometry. J Mass Spectrom.

[20] Olsen JV, Schwartz JC, Griep-Raming J, Nielsen ML, Damoc E, Denisov E, Lange O, Remes P, Taylor D, Splendore M, Wouters ER, Senko M, Makarov A, Mann M, Horning S (2009). A dual pressure linear ion trap Orbitrap instrument with very high sequencing speed. Mol Cell Proteomics.

[21] Michalski A, Damoc E, Lange O, Denisov E, Nolting D, Müller M, Viner R, Schwartz J, Remes P, Belford M, Dunyach JJ, Cox J, Horning S, Mann M, Makarov A (2012). Ultra high resolution linear ion trap Orbitrap mass spectrometer (Orbitrap Elite) facilitates top down LC MS/MS and versatile peptide fragmentation modes. Mol Cell Proteomics.

[22] Cox J, Mann M (2008). MaxQuant enables high peptide identification rates, individualized ppb-range mass accuracies and proteome-wide protein quantification. Nat Biotechnol.

[23] Shao S, Guo T, Aebersold R (2015). Mass spectrometry-based proteomic quest for diabetes biomarkers. Biochim Biophys Acta.

[24] Sleno L, Volmer DA (2004). Ion activation methods for tandem mass spectrometry. J Mass Spectrom.

[25] Wells JM, McLuckey SA (2005). Collision-induced dissociation (CID) of peptides and proteins. Methods Enzymol.

[26] Schroeder MJ, Shabanowitz J, Schwartz JC, Hunt DF, Coon JJ (2004). A neutral loss activation method for improved phosphopeptide sequence analysis by quadrupole ion trap mass spectrometry. Anal Chem..

[27] Olsen JV, Macek B, Lange O, Makarov A, Horning S, Mann M (2007). Higher-energy C-trap dissociation for peptide modification analysis. Nat Methods.

[28] Zubarev RA, Horn DM, Fridriksson EK, Kelleher NL, Kruger NA, Lewis MA, Carpenter BK, McLafferty FW (2000). Electron capture dissociation for structural characterization of multiply charged protein cations. Anal Chem.

[29] Syka JE, Coon JJ, Schroeder MJ, Shabanowitz J, Hunt DF (2004). Peptide and protein sequence analysis by electron transfer dissociation mass spectrometry. Proc Natl Acad Sci USA.

[30] Kim MS, Pandey A (2012). Electron transfer dissociation mass spectrometry in proteomics. Proteomics.

[31] Chi A, Huttenhower C, Geer LY, Coon JJ, Syka JE, Bai DL, Shabanowitz J, Burke DJ, Troyanskaya OG (2007). Hunt DF Analysis of phosphorylation sites on proteins from *Saccharomyces cerevisiae* by electron transfer dissociation (ETD) mass spectrometry. Proc Natl Acad Sci USA.

[32] Ong SE, Mann M (2005). Mass spectrometry-based proteomics turns quantitative. Nat Chem Biol.

[33] Ong SE, Blagoev B, Kratchmarova I, Kristensen DB, Steen H, Pandey A, Mann M (2002). Stable isotope labeling by amino acids in cell culture, SILAC, as a simple and accurate approach to expression proteomics. Mol Cell Proteomics.

[34] Mann M (2006). Functional and quantitative proteomics using SILAC. Nat Rev Mol Cell Biol.

[35] Geiger T, Cox J, Ostasiewicz P, Wisniewski JR, Mann M (2010). Super-SILAC mix for quantitative proteomics of human tumor tissue. Nat Methods.

[36] Krüger M, Moser M, Ussar S, Thievessen I, Luber CA, Forner F, Schmidt S, Zanivan S, Fässler R, Mann M (2008). SILAC mouse for quantitative proteomics uncovers kindlin-3 as an essential factor for red blood cell function. Cell.

[37] Xu P, Tan H, Duong DM, Yang Y, Kupsco J, Moberg KH, Li H, Jin P, Peng J (2012). Stable isotope labeling with amino acids in Drosophila for quantifying proteins and modifications. J Proteome Res..

[38] Cuomo A, Sanfilippo R, Vaccari T, Bonaldi T (2014). Proteomics meets genetics: SILAC labeling of *Drosophila melanogaster* larvae and cells for in vivo functional studies. Methods Mol Biol..

[39] Poitout V, Olson LK, Robertson RP (1996). Insulin-secreting cell lines:classification, characteristics and potential applications. Diabetes Metab..

[40] Krijgsveld J, Ketting RF, Mahmoudi T, Johansen J, Artal-Sanz M, Verrijzer CP, Plasterk RH, Heck AJ (2003). Metabolic labeling of *C. elegans* and *D. melanogaster* for quantitative proteomics. Nat Biotechnol.

[41] Nielsen ML, Vermeulen M, Bonaldi T, Cox J, Moroder L, Mann M (2008). Iodoacetamide-induced artifact mimics ubiquitination in mass spectrometry. Nat Methods.

[42] Hsu JL, Huang SY, Chow NH, Chen SH (2003). Stable-isotope dimethyl labeling for quantitative proteomics. Anal Chem.

[43] Boersema PJ, Raijmakers R, Lemeer S, Mohammed S, Heck AJ (2009). Multiplex peptide stable isotope dimethyl labeling for quantitative proteomics. Nat Protoc.

[44] Ross PL, Huang YN, Marchese JN, Williamson B, Parker K, Hattan S, Khainovski N, Pillai S, Dey S, Daniels S, Purkayastha S, Juhasz P, Martin S, Bartlet-Jones M, He F, Jacobson A, Pappin DJ (2004). Multiplexed protein quantitation in *Saccharomyces cerevisiae* using amine-reactive isobaric tagging reagents. Mol Cell Proteomics.

[45] Ow SY, Salim M, Noirel J, Evans C, Rehman I, Wright PC (2009). iTRAQ underestimation in simple and complex mixtures: “the good, the bad and the ugly”. J Proteome Res.

[46] Zhang Y, Askenazi M, Jiang J, Luckey CJ, Griffin JD, Marto JA (2010). A robust error model for iTRAQ quantification reveals divergent signaling between oncogenic FLT3 mutants in acute myeloid leukemia. Mol Cell Proteomics.

[47] Shiio Y, Aebersold R (2006). Quantitative proteome analysis using isotope-coded affinity tags and mass spectrometry. Nat Protoc..

[48] Wong JW, Cagney G (2010). An overview of label-free quantitation methods in proteomics by mass spectrometry. Methods Mol Biol.

[49] Mueller LN, Brusniak MY, Mani DR, Aebersold R (2008). An assessment of software solutions for the analysis of mass spectrometry based quantitative proteomics data. J Proteome Res.

[50] Luber CA, Cox J, Lauterbach H, Fancke B, Selbach M, Tschopp J, Akira S, Wiegand M, Hochrein H, O’Keeffe M, Mann M (2010). Quantitative proteomics reveals subset-specific viral recognition in dendritic cells. Immunity.

[51] Cox J, Hein MY, Luber CA, Paron I, Nagaraj N, Mann M (2014). Accurate proteome-wide label free quantification by delayed normalization and maximal peptide ratio extraction, termed MaxLFQ. Mol Cell Proteomics.

[52] Kirkpatrick DS, Gerber SA, Gygi SP (2005). The absolute quantification strategy: a general procedure for the quantification of proteins and post-translational modifications. Methods.

[53] Hanke S, Besir H, Oesterhelt D, Mann M (2008). Absolute SILAC for accurate quantitation of proteins in complex mixtures down to the attomole level. J Proteome Res..

[54] Kumar C, Mann M (2009). Bioinformatics analysis of mass spectrometry-based proteomics data sets. FEBS Lett.

[55] Ludwig C, Gillet L, Rosenberger G, Amon S, Collins BC, Aebersold R (2018). Data-independent acquisition-based SWATH-MS for quantitative proteomics: a tutorial. Mol Syst Biol..

[56] Ashburner M, Ball CA, Blake JA, Botstein D, Butler H, Cherry JM, Davis AP, Dolinski K, Dwight SS, Eppig JT, Harris MA, Hill DP, Issel-Tarver L, Kasarskis A, Lewis S, Matese JC, Richardson JE, Ringwald M, Rubin GM, Sherlock G (2000). Gene ontology: tool for the unification of biology. The gene ontology consortium. Nat Genet..

[57] Kanehisa M, Goto S, Kawashima S, Okuno Y, Hattori M (2004). The KEGG resource for deciphering the genome. Nucleic Acids Res.

[58] Andersson L, Porath J (1986). Isolation of phosphoproteins by immobilized metal (Fe3 +) affinity chromatography. Anal Biochem.

[59] Neville DC, Rozanas CR, Price EM, Gruis DB, Verkman AS, Townsend RR (1997). Evidence for phosphorylation of serine 753 in CFTR using a novel metal-ion affinity resin and matrix-assisted laser desorption mass spectrometry. Protein Sci.

[60] Zhou H, Ye M, Dong J, Han G, Jiang X, Wu R, Zou H (2008). Specific phosphopeptide enrichment with immobilized titanium ion affinity chromatography adsorbent for phosphoproteome analysis. J Proteome Res.

[61] Posewitz MC, Tempst P (1999). Immobilized gallium (III) affinity chromatography of phosphopeptides. Anal Chem.

[62] Feng S, Ye M, Zhou H, Jiang X, Jiang X, Zou H, Gong B (2007). Immobilized zirconium ion affinity chromatography for specific enrichment of phosphopeptides in phosphoproteome analysis. Mol Cell Proteomics.

[63] Larsen MR, Thingholm TE, Jensen ON, Roepstorff P, Jorgensen TJ (2005). Highly selective enrichment of phosphorylated peptides from peptide mixtures using titanium dioxide microcolumns. Mol Cell Proteomics.

[64] McNulty DE, Annan RS (2008). Hydrophilic interaction chromatography reduces the complexity of the phosphoproteome and improves global phosphopeptide isolation and detection. Mol Cell Proteomics.

[65] Beausoleil SA, Jedrychowski M, Schwartz D, Elias JE, Villén J, Li J, Cohn MA, Cantley LC, Gygi SP (2004). Large-scale characterization of HeLa cell nuclear phosphoproteins. Proc Natl Acad Sci USA.

[66] De Corte V, Demol H, Goethals M, Van Damme J, Gettemans J, Vandekerckhove J (1999). Identification of Tyr438 as the major in vitro c-Src phosphorylation site in human gelsolin: a mass spectrometric approach. Protein Sci.

[67] Rush J, Moritz A, Lee KA, Guo A, Goss VL, Spek EJ, Zhang H, Zha XM, Polakiewicz RD, Comb MJ (2005). Immunoaffinity profiling of tyrosine phosphorylation in cancer cells. Nat Biotechnol.

[68] Rikova K, Guo A, Zeng Q, Possemato A, Yu J, Haack H, Nardone J, Lee K, Reeves C, Li Y, Hu Y, Tan Z, Stokes M, Sullivan L, Mitchell J, Wetzel R, Macneill J, Ren JM, Yuan J, Bakalarski CE, Villen J, Kornhauser JM, Smith B, Li D, Zhou X, Gygi SP, Gu TL, Polakiewicz RD, Rush J, Comb MJ (2007). Global survey of phosphotyrosine signaling identifies oncogenic kinases in lung cancer. Cell.

[69] Boersema PJ, Foong LY, Ding VM, Lemeer S, van Breukelen B, Philp R, Boekhorst J, Snel B, den Hertog J, Choo AB, Heck AJ (2010). In-depth qualitative and quantitative profiling of tyrosine phosphorylation using a combination of phosphopeptide immunoaffinity purification and stable isotope dimethyl labeling. Mol Cell Proteomics.

[70] Hunter T, Sefton BM (1980). Transforming gene-product of rous-sarcoma virus phosphorylates tyrosine. Proc Natl Acad Sci USA.

[71] Szabat M, Lynn FC, Hoffman BG, Kieffer TJ, Allan DW, Johnson JD (2012). Maintenance of β-cell maturity and plasticity in the adult pancreas: developmental biology concepts in adult physiology. Diabetes.

[72] Weir GC, Aguayo-Mazzucato C, Bonner-Weir S (2013). β-Cell dedifferentiation in diabetes is important, but what is it?. Islets..

[73] Szabat M, Page MM, Panzhinskiy E, Skovsø S, Mojibian M, Fernandez-Tajes J, Bruin JE, Bround MJ, Lee JT, Xu EE, Taghizadeh F, O’Dwyer S, Van de Bunt M, Moon KM, Sinha S, Han J, Fan Y, Lynn FC, Trucco M, Borchers CH, Foster LJ, Nislow C, Kieffer TJ, Johnson JD (2016). Reduced insulin production relieves endoplasmic reticulum stress and induces β cell proliferation. Cell Metab.

[74] Li J, Li Q, Tang J, Xia F, Wu J, Zeng R (2015). Quantitative phosphoproteomics revealed glucose-stimulated responses of islet associated with insulin secretion. J Proteome Res..

[75] Humphrey SJ, Yang G, Yang P, Fazakerley DJ, Stockli J, Yang JY, James DE (2013). Dynamic adipocyte phosphoproteome reveals that Akt directly regulates mTORC2. Cell Metab.

[76] Zhang Y, Zhang Y, Yu Y (2017). Global phosphoproteomic analysis of insulin/Akt/mTORC1/S6K signaling in rat hepatocytes. J Proteome Res.

[77] Iwai LK, Benoist C, Mathis D, White FM (2010). Quantitative phosphoproteomic analysis of T cell receptor signaling in diabetes prone and resistant mice. J Proteome Res..

[78] Højlund K, Yi Z, Lefort N, Langlais P, Bowen B, Levin K, Beck-Nielsen H, Mandarino LJ (2010). Human ATP synthase beta is phosphorylated at multiple sites and shows abnormal phosphorylation at specific sites in insulin-resistant muscle. Diabetologia..

[79] Shinoda K, Ohyama K, Hasegawa Y, Chang HY, Ogura M, Sato A, Hong H, Hosono T, Sharp LZ, Scheel DW, Graham M, Ishihama Y, Kajimura S (2015). Phosphoproteomics identifies CK2 as a negative regulator of beige adipocyte thermogenesis and energy expenditure. Cell Metab.

[80] Chursa U, Nuñez-Durán E, Cansby E, Amrutkar M, Sütt S, Ståhlman M, Olsson BM, Borén J, Johansson ME, Bäckhed F, Johansson BR, Sihlbom C, Mahlapuu M (2017). Overexpression of protein kinase STK25 in mice exacerbates ectopic lipid accumulation, mitochondrial dysfunction and insulin resistance in skeletal muscle. Diabetologia.

[81] Yang X, Ongusaha PP, Miles PD, Havstad JC, Zhang F, So WV, Kudlow JE, Michell RH, Olefsky JM, Field SJ, Evans RM (2008). Phosphoinositide signalling links O-GlcNAc transferase to insulin resistance. Nature.

[82] Catalina MI, Koeleman CAM, Deelder AM, Wuhrer M (2007). Electron transfer dissociation of N-glycopeptides: loss of the entire N-glycosylated asparagine sidechain. Rapid Commun Mass Spectrom.

[83] Wang Z, Udeshi ND, O’Malley M, Shabanowitz J, Hunt DF, Hart GW (2010). Enrichment and site mapping of O-linked N-acetylglucosamine by a combination of chemical/enzymatic tagging, photochemical cleavage, and electron transfer dissociation mass spectrometry. Mol Cell Proteomics.

[84] Yang W, Shah P, Hu Y, Toghi Eshghi S, Sun S, Liu Y, Zhang H (2017). Comparison of enrichment methods for intact N- and O-linked glycopeptides using strong anion exchange and hydrophilic interaction liquid chromatography. Anal Chem.

[85] Ito S, Hayama K, Hirabayashi J (2009). Enrichment strategies for glycopeptides. Methods Mol Biol.

[86] Bunkenborg J, Pilch BJ, Podtelejnikov AV, Wisniewski JR (2004). Screening for N-glycosylated proteins by liquid chromatography mass spectrometry. Proteomics.

[87] Zhang H, Li XJ, Martin DB, Aebersold R (2003). Identification and quantification of N-linked glycoproteins using hydrazide chemistry, stable isotope labeling and mass spectrometry. Nat Biotechnol.

[88] Fang C, Xiong Z, Qin H, Huang G, Liu J, Ye M, Feng S, Zou H (2014). One-pot synthesis of magnetic colloidal nanocrystal clusters coated with chitosan for selective enrichment of glycopeptides. Anal ChimActa..

[89] Pohlentz G, Marx K, Mormann M (2016). Characterization of protein N-glycosylation by analysis of ZIC-HILIC-enriched intact proteolytic glycopeptides. Methods Mol Biol.

[90] Parker BL, Thaysen-Andersen M, Fazakerley DJ, Holliday M, Packer NH, James DE (2016). Terminal galactosylation and sialylation switching on membrane glycoproteins upon TNF-alpha-induced insulin resistance in adipocytes. Mol Cell Proteomics..

[91] Lim JM, Wollaston-Hayden EE, Teo CF, Hausman D, Wells L (2014). Quantitative secretome and glycome of primary human adipocytes during insulin resistance. Clin Proteomics..

[92] Comer FI, Vosseller K, Wells L, Accavitti MA, Hart GW (2001). Characterization of a mouse monoclonal antibody specific for O-linked N-acetylglucosamine. Anal Biochem..

[93] Teo CF, Ingale S, Wolfert MA, Elsayed GA, Not LG, Chatham JC, Wells L, Boons GJ (2010). Glycopeptide-specific monoclonal antibodies suggest new roles for O-GlcNAc. Nat Chem Biol.

[94] Zachara NE, Vosseller K, Hart GW. Detection and analysis of proteins modified by O linked N-acetylglucosamine. Curr Protoc Protein Sci. 2011; Chapter 12:Unit12.8. 10.1002/0471140864.ps1208s66 PMC3349994 22045558

[95] Kamemura K, Hayes BK, Comer FI, Hart GW (2002). Dynamic interplay between O-glycosylation and O-phosphorylation of nucleocytoplasmic proteins: alternative glycosylation/phosphorylation of THR-58, a known mutational hot spot of c-Myc in lymphomas, is regulated by mitogens. J Biol Chem.

[96] Pathak S, Borodkin SA, Osama A, Campbell DG, Ibrahim A, Daan MF, van Aalten DM (2012). OGlcNAcylation of TAB 1 modulates TAK1-mediated cytokine release. EMBO J.

[97] Yuzwa SA, Yadav AK, Skorobogatko Y, Clark T, Vosseller K, Vocadlo DJ (2011). Mapping O-GlcNAc modification sites on tau and generation of a site-specific OGlcNAc tau antibody. Amino Acids.

[98] Vosseller K, Trinidad JC, Chalkley RJ, Specht CG, Thalhammer A, Lynn AJ, Snedecor JO, Guan S, Medzihradszky KF, Maltby DA, Schoepfer R, Burlingame AL (2006). O-linked Nacetylglucosamine proteomics of postsynaptic density preparations using lectin weak affinity chromatography and mass spectrometry. Mol Cell Proteomics.

[99] Trinidad JC, Barkan DT, Gulledge BF, Thalhammer A, Sali A, Schoepfer R, Burlingame AL (2012). Global identification and characterization of both O-GlcNAcylation and phosphorylation at the murine synapse. Mol Cell Proteomics.

[100] Hahne H, Sobotzki N, Nyberg T, Helm D, Borodkin VS, van Aalten DM, Agnew B, Kuster B (2013). Proteome wide purification and identification of O-GlcNAc-modified proteins using click chemistry and mass spectrometry. J Proteome Res.

[101] Wu ZL, Robey MT, Tatge T, Lin C, Leymarie N, Zou Y, Zaia J (2014). Detecting O-GlcNAc using in vitro sulfation. Glycobiology.

[102] Ma J, Hart GW (2013). Protein O-GlcNAcylation in diabetes and diabetic complications. Expert Rev Proteomics..

[103] Park J, Kwon H, Kang Y, Kim Y (2007). Proteomic analysis of O-GlcNAc modifications derived from streptozotocin and glucosamine induced beta-cell apoptosis. J Biochem Mol Biol..

[104] Akimoto Y, Miura Y, Toda T, Wolfert MA, Wells L, Boons GJ, Hart GW, Endo T, Kawakami H (2011). Morphological changes in diabetic kidney are associated with increased O-GlcNAcylation of cytoskeletal proteins including α-actinin 4. Clin Proteomics.

[105] Wright JN, Collins HE, Wende AR, Chatham JC (2017). O-GlcNAcylation and cardiovascular disease. Biochem Soc Trans.

[106] Erickson JR, Pereira L, Wang L, Han G, Ferguson A, Dao K, Copeland RJ, Despa F, Hart GW, Ripplinger CM, Bers DM (2013). Diabetic hyperglycaemia activates CaMKII and arrhythmias by O-linked glycosylation. Nature.

[107] Ramirez-Correa GA, Ma J, Slawson C, Zeidan Q, Lugo-Fagundo NS, Xu M, Shen X, Gao WD, Caceres V, Chakir K, DeVine L, Cole RN, Marchionni L, Paolocci N, Hart GW, Murphy AM (2015). Removal of abnormal myofilament O-GlcNAcylation restores Ca2 + sensitivity in diabetic cardiac muscle. Diabetes.

[108] Banerjee PS, Ma J, Hart GW (2015). Diabetes-associated dysregulation of O-GlcNAcylation in rat cardiac mitochondria. Proc Natl Acad Sci USA.

[109] Ball LE, Berkaw MN, Buse MG (2006). Identification of the major site of O-linked beta-N-acetylglucosamine modification in the C terminus of insulin receptor substrate-1. Mol Cell Proteomics.

[110] Klein AL, Berkaw MN, Buse MG, Ball LE (2009). O-linked N-acetylglucosamine modification of insulin receptor substrate-1 occurs in close proximity to multiple SH2 domain binding motifs. Mol Cell Proteomics.

[111] Dehennaut V, Lefebvre T (2013). Proteomics and PUGNAcity will overcome questioning of insulin resistance induction by nonselective inhibition of O-GlcNAcase. Proteomics.

[112] Vosseller K, Wells L, Lane MD, Hart GW (2002). Elevated nucleocytoplasmic glycosylation by O-GlcNAc results in insulin resistance associated with defects in Akt activation in 3T3-L1 adipocytes. Proc Natl Acad Sci USA.

[113] Arias EB, Kim J, Cartee GD (2004). Prolonged incubation in PUGNAc results in increased protein O linked glycosylation and insulin resistance in rat skeletal muscle. Diabetes.

[114] Lee JE, Park JH, Moon PG (2013). Baek MC Identification of differentially expressed proteins by treatment with PUGNAc in 3T3-L1 adipocytes through analysis of ATP binding proteome. Proteomics.

[115] Housley MP, Rodgers JT, Udeshi ND, Kelly TJ, Shabanowitz J, Hunt DF, Puigserver P, Hart GW (2008). O-GlcNAc regulates FoxO activation in response to glucose. J Biol Chem.

[116] Dentin R, Hedrick S, Xie J, Yates J, Montminy M (2008). Hepatic glucose sensing via the CREB coactivator CRTC2. Science.

[117] Ruan HB, Han X, Li MD, Singh JP, Qian K, Azarhoush S, Zhao L, Bennett AM, Samuel VT, Wu J, Yates JR, Yang X (2012). O-GlcNAc transferase/host cell factor C1 complex regulates gluconeogenesis by modulating PGC-1alpha stability. Cell Metab.

[118] Wang Z, Park K, Comer F, Hsieh-Wilson LC, Saudek CD, Hart GW (2009). Site-specific GlcNAcylation of human erythrocyte proteins: potential biomarker(s) for diabetes. Diabetes.

[119] Kaisaki PJ, Otto GW, McGouran JF, Toubal A, Argoud K, Waller-Evans H, Finlay C, Caldérari S, Bihoreau MT, Kessler BM, Gauguier D, Mott R (2014). Genetic control of differential acetylation in diabetic rats. PLoS ONE.

[120] Kadiyala CS, Zheng L, Du Y, Yohannes E, Kao HY, Miyagi M, Kern TS (2012). Acetylation of retinal histones in diabetes increases inflammatory proteins: effects of minocycline and manipulation of histone acetyltransferase (HAT) and histone deacetylase (HDAC). J Biol Chem.

[121] Alrob OA, Sankaralingam S, Ma C, Wagg CS, Fillmore N, Jaswal JS, Sack MN, Lehner R, Gupta MP, Michelakis ED, Padwal RS, Johnstone DE, Sharma AM, Lopaschuk GD (2014). Obesity-induced lysine acetylation increases cardiac fatty acid oxidation and impairs insulin signalling. Cardiovasc Res.

[122] Vazquez EJ, Berthiaume JM, Kamath V, Achike O, Buchanan E, Montano MM, Chandler MP, Miyagi M, Rosca MG (2015). Mitochondrial complex I defect and increased fatty acid oxidation enhance protein lysine acetylation in the diabetic heart. Cardiovasc Res..

[123] Hölper S, Nolte H, Bober E, Braun T, Krüger M (2015). Dissection of metabolic pathways in the Db/Db mouse model by integrative proteome and acetylome analysis. Mol Biosyst..

[124] Zhang Q, Monroe ME, Schepmoes AA, Clauss TR, Gritsenko MA, Meng D, Petyuk VA, Smith RD, Metz TO (2011). Comprehensive identification of glycated peptides and their glycation motifs in plasma and erythrocytes of control and diabetic subjects. J Proteome Res.

[125] Ott C, Jacobs K, Haucke E, Navarrete Santos A, Grune T, Simm A (2014). Role of advanced glycation end products in cellular signaling. Redox Biol..

[126] Li BY, Li XL, Cai Q, Gao HQ, Cheng M, Zhang JH, Wang JF, Yu F, Zhou RH (2011). Induction of lactadherin mediates the apoptosis of endothelial cells in response to advanced glycation end products and protective effects of grape seed procyanidin B2 and resveratrol. Apoptosis.

[127] Walsh G, Jefferis R (2006). Post-translational modifications in the context of therapeutic proteins. Nat Biotechnol.

[128] Mischak H, Ioannidis JP, Argiles A, Attwood TK, Bongcam-Rudloff E, Broenstrup M, Charonis A, Chrousos GP, Delles C, Dominiczak A, Dylag T, Ehrich J, Egido J, Findeisen P, Jankowski J, Johnson RW, Julien BA, Lankisch T, Leung HY, Maahs D, Magni F, Manns MP, Manolis E, Mayer G, Navis G, Novak J, Ortiz A, Persson F, Peter K, Riese HH, Rossing P, Sattar N, Spasovski G, Thongboonkerd V, Vanholder R, Schanstra JP, Vlahou A (2012). Implementation of proteomic biomarkers: making it work. Eur J Clin Invest.

[129] Choudhary C, Mann M (2010). Decoding signalling networks by mass spectrometry-based proteomics. Nat Rev Mol Cell Biol.

